# Subcellular Specialization of Mitochondrial Form and Function in Skeletal Muscle Cells

**DOI:** 10.3389/fcell.2021.757305

**Published:** 2021-10-15

**Authors:** T. Bradley Willingham, Peter T. Ajayi, Brian Glancy

**Affiliations:** ^1^National Heart, Lung and Blood Institute, National Institutes of Health, Bethesda, MD, United States; ^2^National Institute of Arthritis and Musculoskeletal and Skin Diseases, National Institutes of Health, Bethesda, MD, United States

**Keywords:** paravascular mitochondria, subsarcolemmal mitochondria, intermyofibrillar mitochondria, organelle interactions, paranuclear mitochondria, mitochondrial respiration, bioenergetics, mitochondrial connectivity

## Abstract

Across different cell types and within single cells, mitochondria are heterogeneous in form and function. In skeletal muscle cells, morphologically and functionally distinct subpopulations of mitochondria have been identified, but the mechanisms by which the subcellular specialization of mitochondria contributes to energy homeostasis in working muscles remains unclear. Here, we discuss the current data regarding mitochondrial heterogeneity in skeletal muscle cells and highlight potential new lines of inquiry that have emerged due to advancements in cellular imaging technologies.

## Introduction

**FIGURE 1:**
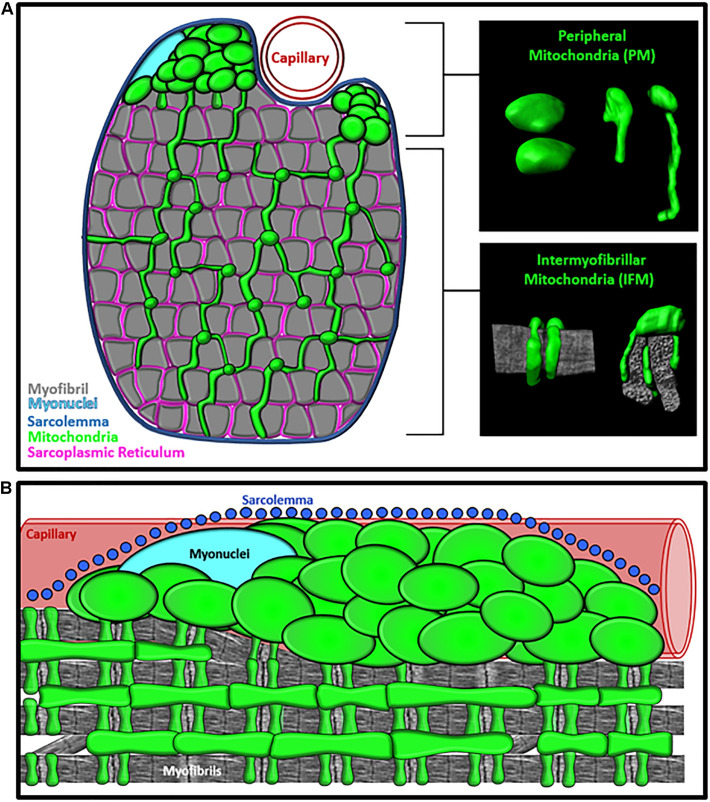
Specialization of Mitochondrial Morphology in Skeletal Muscle. **(A)** Cross-section diagram of muscle cell showing the morphologically distinct peripherally-located mitochondria (PM) and intermyofibrillar mitochondria (IFM) subpopulations. Inserts provide examples of 3D morphology of PM and IFM. Mitochondria (green) and Myofibrils (gray). **(B)** Longitudinal view of muscle cell showing the subcellular location of PM and IFM relative to the sarcolemma (blue), capillary (red), Myonuclei (cyan), and myofibrils (gray).

Skeletal muscle cells function to convert biological fuels to mechanical force and are therefore critical to many physiological functions, including movement and metabolism. During sustained muscle contractions, the process of cellular energy transduction is largely mediated by the energy-converting function of the mitochondria and the force-generating capacity of the myofibrils. Specifically, mitochondria convert biological fuels to the high energy molecule adenosine triphosphate (ATP) through oxidative phosphorylation, and ATP is used by the myofibrillar ATPase to generate force during contraction. Although the vast majority of muscle cell volume is dedicated to the myofibrillar matrix (Willingham et al., 2020), mammalian skeletal muscles can be 2–10% mitochondria by volume (Bleck et al., 2018), and muscle cells with higher mitochondrial content have greater energy-converting capacity (Holloszy, 1967; Schwerzmann et al., 1989; Ørtenblad et al., 2018). Like most cell types, not all mitochondria within the muscle cell are the same, and mitochondrial form and function can vary widely within a single muscle cell (Johnson et al., 2007; Lewis et al., 2016; Valm et al., 2017; Benador et al., 2018). For the past half-century, experimental studies have consistently demonstrated that muscle cells contain structurally and functionally distinct subpopulations of mitochondria based on their proximity to the myofibrils, nuclei, capillaries, and cell membrane (Palmer et al., 1977, 1985; Cogswell et al., 1993; Takahashi and Hood, 1996; Rothstein et al., 2005; Glancy et al., 2014, 2015, 2018; Figure 1). However, the defining characteristics (e.g., molecular markers) of mitochondrial subpopulations in skeletal muscle cells remain elusive, and the mechanisms by which the subcellular specialization of mitochondria contributes to energy homeostasis in working muscles remains unclear. Here, we review current data regarding mitochondrial heterogeneity in skeletal muscle cells and discuss potential implications for the subcellular specialization of mitochondrial form and function. Particularly, we focus on how the development of subcellular imaging technologies expands our capacity to simultaneously evaluate muscle structure and function and highlight potential new lines of inquiry regarding the subcellular specialization of mitochondria.

## Subcellular Specialization of Mitochondrial Structure

**FIGURE 2:**
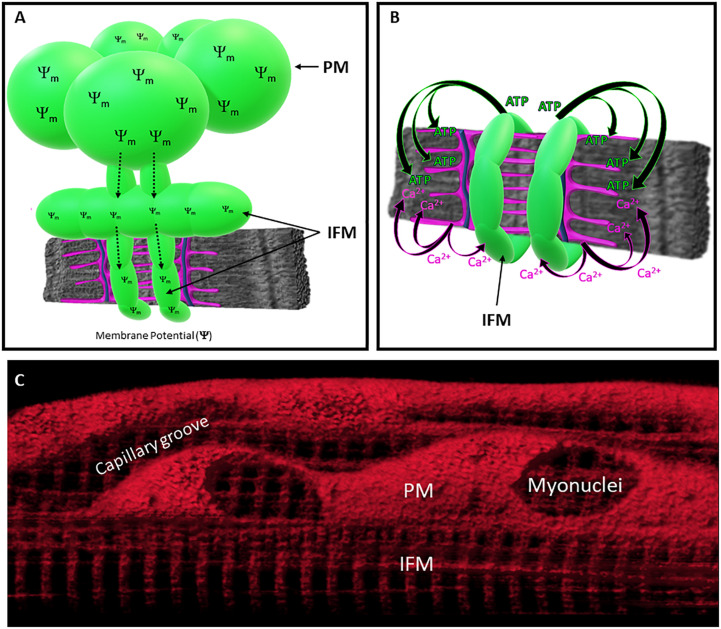
Specialization of Mitochondrial Function across Subcellular Regions. **(A)** Membrane potential generated in PM can be distributed throughout the IFM network through mitochondrial connectivity. **(B)** IFM directly supports myofibrillar contraction by generating ATP. Coordination between myofibrillar contraction and IFM energy conversion are mediated by Ca^2+^ release from the sarcoplasmic reticulum (SR). Mitochondria (green), SR (magenta), *t*-tubule (blue), and myofibril (gray). **(C)** Mitochondrial membrane potential distributed throughout the mitochondrial network of living skeletal muscle cell.

Within the interior region of the muscle cell, the delicate interaction between the mitochondria and myofibrils supports precise coordination among the metabolic and mechanical arms of the muscle cellular energy distribution system. The intrafibrillar mitochondria (IFMs) form complex, elongated shapes (Hoppeler et al., 1973a; Picard et al., 2013a,b, 2015; Glancy et al., 2015; Bleck et al., 2018) with branches that extend between the crevices of the myofibrillar matrix and wrap around the I-band of the sarcomere (Hoppeler et al., 1973a; Picard et al., 2013a,b, 2015; Glancy et al., 2015; Bleck et al., 2018; Vincent et al., 2019; Figure 1). In human muscles, mitochondria entrenched within the intermyofibrillar spaces are nearly double the aspect ratio compared to mitochondria located in the periphery, resulting in morphologies with a high surface area to volume ratio (Picard et al., 2013b; Vincent et al., 2019). This high surface area to volume ratio facilitates the rapid diffusion of high-energy ATP molecules from the mitochondria to the myofibrillar ATPase within this region of the cell (Figure 2). During muscle contraction, the metabolic function of the mitochondria and contractile function of myofibrils are also mediated by Ca^2+^ signaling from the sarcoplasmic reticulum (SR) (Figure 2), so IFMs also utilize their available surface area to form functional contact-sites (i.e., membranes within 30 nm) with the SR (Eisner et al., 2013; Tubbs et al., 2018; Figure 1). Studies using high-resolution 3D electron microscopy to evaluate connectivity in mouse muscles across several striated muscle types have shown that nearly all IFM (>97%) contact the SR. Moreover, this same work also found that ∼20% of the IFM within oxidative muscle cells directly contact lipid droplets while no lipid droplets were found in glycolytic muscle cells (Bleck et al., 2018). Although these data indicate that mitochondria-lipid interactions may contribute to unique mitochondrial function across different cell types, information regarding differences in mitochondria-organelle interactivity between mitochondrial subgroups remains limited. Studies evaluating the spatial distribution of lipids in human muscle cells have shown that lipid droplets preferentially localize to the interfibrillar region of the cell (Nielsen et al., 2010), but it is still unclear how the subcellular distribution of mitochondria-lipid droplet interactions might contribute to the regional specialization of mitochondrial function.

Mitochondria also directly interact with other mitochondria, and studies have shown that IFM connect together to form networks that function to distribute energy throughout the entire cell (Bakeeva et al., 1978; Luo et al., 2013; Picard et al., 2013a,b, 2015; Eisner et al., 2014; Glancy et al., 2015, 2018; Bleck et al., 2018; Vincent et al., 2019; Figure 2). Mitochondria-mitochondria contact sites appear as electron-dense junctions (Glancy et al., 2015; Picard et al., 2015), and high-resolution electron microscopy images of these contact sites have shown that portions of the adjacent outer membranes of these connected mitochondria are in direct contact (<1 nm separation) (Bakeeva et al., 1978; Picard et al., 2013a,b, 2015; Glancy et al., 2015, 2018; Bleck et al., 2018; Vincent et al., 2019) and often exhibit *trans-*mitochondrial alignment of cristae (Picard et al., 2015). Thus, structural capabilities exist within muscle cells for effective energy distribution, challenging the long-held theory that facilitated diffusion is the primary means of distributing intracellular energy within muscles (Wittenberg, 1970; Bessman and Geiger, 1981). Indeed, the phosphocreatine (PCr) shuttle system may also function to facilitate the subcellular distribution of high energy phosphates within muscle cells (Bessman and Geiger, 1981), though mice lacking creatine and creatine kinase do not exhibit robust changes in muscle function (van Deursen et al., 1993; Lygate et al., 2013). Alternatively, live cell imaging studies have demonstrated that electrically coupled mitochondria can transmit membrane potential greater than 10 μm across the intermyofibrillar space (Bleck et al., 2018), providing an additional mechanism for the rapid distribution of potential energy throughout the subcellular environment. Moreover, when comparing the 3D configuration of mitochondrial networks in different striated muscle cell types, mitochondrial connectivity and overall network configuration are cell-type specific, such that glycolytic, oxidative, and cardiac muscles have perpendicular, grid-like, and parallel orientations, respectively (Bleck et al., 2018; Willingham et al., 2019). These findings suggest that the degree of connectivity and spatial distribution within the IFM network are optimized to meet specific regional demands within different muscle types.

Early imaging studies in muscle cell biology used 2D electron microscopy (Romanul, 1964, 1965; Bubenzer, 1966; Gauthier and Padykula, 1966) to reveal a second, morphologically-distinct, subgroup of peripherally-located mitochondria (PM). In the peripheral regions of the muscle cell, pools of globular mitochondria were found clustered together in the intracellular space between the sarcolemma and myofibrils (Romanul, 1964, 1965; Bubenzer, 1966; Gauthier and Padykula, 1966; Hoppeler et al., 1973a; Figure 1). Not long after these initial observations, studies by Bubenzer (1966), and later Bakeeva et al. (1978), demonstrated that PM extend into the intermyofibrillar space and form direct connections with the IFM. These authors proposed that mitochondria connect across all subcellular regions to work together as an “effective mechanism of energy transport” (Bakeeva et al., 1978). Under this model, the morphologically distinct PM subgroups are functionally specialized to support the intermyofibrillar regions of the mitochondrial networks (Figure 2). When comparing the morphology of individual mitochondria within the IFM and PM subgroups, PM are larger and less branched than IFM (Bakeeva et al., 1978; Picard et al., 2013a,b, 2015; Glancy et al., 2015, 2018; Bleck et al., 2018; Vincent et al., 2019), dedicating more of their volume to cristae and matrix, providing greater structural capacity for energy conversion. Studies in creatine kinase (CK) knockout mice have reported that PM subgroups increase in response to inhibiting the PCr shuttle system suggesting that increasing PM volume may compensate for the loss of the diffusion-mediated energy distribution (Novotova et al., 2006) though these data may simply reflect a fiber type shift toward a more oxidative phenotype. High-resolution imaging of human and mouse muscles have also shown that PM form frequent connections with other mitochondria, but few studies have directly quantified mitochondrial-organelle interactions of PM in skeletal muscle cells (Picard et al., 2013a,b; Bleck et al., 2018). Thus, it is currently unclear how differences in organelle interactivity may contribute to structural mechanisms of mitochondrial specialization.

The functional specificity of PM may also be related to their subcellular location. Historically, PM have been classified as subsarcolemmal mitochondria (SSM) (Muller, 1976; Palmer et al., 1977, 1985; Elander et al., 1985; Cogswell et al., 1993; Manneschi and Federico, 1995; Takahashi and Hood, 1996), but much of the subsarcolemmal space within a muscle cell is densely packed with myofibrils rather than mitochondria (Figure 1). More recent studies (Rothstein et al., 2005; Glancy et al., 2014, 2015, 2018) have demonstrated that peripherally-located subgroups are essentially “paravascular” mitochondria (PVM) or “paranuclear” mitochondria (PNM) that specifically localize to the intracellular space surrounding embedded capillaries and nuclei (Rothstein et al., 2005; Glancy et al., 2014, 2015; Figure 1). These high-resolution 3D imaging studies revealed that capillaries favorably embed into oxidative muscle cells and that 50% of capillaries located near oxidative muscle cells had a large proportion of their circumference (≥50%) embedded into the sarcolemma of muscle cell (Glancy et al., 2014). Thus, PVM subgroups were found predominantly within the more metabolically active muscle cells (oxidative fiber types) and may indeed provide additional metabolic support to the high energy demands generated within the intermyofibrillar regions of these cells (Figure 2). Moreover, clusters of PNM (Rothstein et al., 2005; Glancy et al., 2014) were also found in the intracellular space flanking nuclei, suggesting that PM subgroups may indiscriminately fill in any myofibrillar void caused by the presence of embedded capillaries and nuclei (Glancy et al., 2014). Therefore, the available research suggests that mitochondria subpopulations that have been historically associated with the sarcolemma are actually localized to other cellular structures that displace myofibrils at the periphery of the cell. However, limited studies exist that explicitly delineate the morphological or biochemical differences in mitochondria associated with either capillaries or nuclei, and future work is needed to establish the functional implications of peripheral mitochondrial distribution in muscle cells.

## Subcellular Specialization of Mitochondrial Function

While the primary function of the IFM is somewhat intuitive when considering their intricate spatial relationships with the energy-hungry myofibrils, it is unclear why evolution would favor the separate subgroups of morphologically distinct mitochondria found in clusters near the cell boundary. As noted above, greater mitochondrial volume observed near the cell boundary supports greater energy conversion than the smaller mitochondria residing within the intermyofibrillar spaces, and therefore, some investigators have postulated that PM function to directly support the IFM by generating and distributing energy. As early as the 1960s, Romanul and colleagues found mitochondrial oxidative enzyme activity to be higher in mitochondria located adjacent to capillaries compared to mitochondria within the interior regions of the muscle cell (Romanul, 1964, 1965). Undoubtedly, oxygen is a primary substrate for mitochondrial respiration, and it may be beneficial for mitochondria to crowd the intracellular space near the capillary oxygen supply. However, previous experiments have been limited in their ability to evaluate oxygen kinetics and/or mitochondrial function with subcellular resolution, and therefore, the relationship between PVM function and their proximity to the capillary remains unclear.

To explore the subcellular specialization of mitochondrial function within skeletal muscle cells, investigators have established techniques that aim to specifically isolate intermyofibrillar mitochondria from the total mitochondria pool within muscles. These techniques typically incorporate a tiered purification method that separates mitochondria according to their degree of interactivity with contractile proteins. Mitochondria isolated from homogenized muscle tissues before protease treatment are considered to have less interaction with contractile proteins and are therefore classified as SSM, whereas mitochondria isolated after digestion are classified as IFM. Although this approach has been implemented in many studies and models (Palmer et al., 1977; Krieger et al., 1980; Cogswell et al., 1993), experiments directly comparing the protein composition and function between subgroups of isolated mitochondria have produced conflicting results (Krieger et al., 1980; Elander et al., 1985; Cogswell et al., 1993; Manneschi and Federico, 1995; Takahashi and Hood, 1996; Jimenez et al., 2002; Adhihetty et al., 2005; Mollica et al., 2006). In several early experiments using protease to separately isolate the IFM and SSM subgroups in rodent skeletal muscle, it was determined that IFM have higher mitochondrial respiration enzyme activities and greater capacity to metabolize oxygen compared to SSM (Krieger et al., 1980; Cogswell et al., 1993; Adhihetty et al., 2005). Furthermore, subsequent work found that IFM import fuels, such as malate, at a 3–4-fold greater rate than SSM (Takahashi and Hood, 1996). While these initial observations indicate that mitochondrial metabolic capacity may be impacted by subcellular localization, experiments using isolated IFM and SSM from human muscle biopsies found oxygen consumption rates similar between the groups (Elander et al., 1985; Fischer et al., 1985). Indeed, some aspects of mitochondrial specialization may be influenced by species-specific changes in cellular ultrastructure and physiological demand. For example although PM and IFM subpopulations are consistently observed in humans, mice, and shrews, mitochondria within the shrew oxidative muscle fibers are primarily aligned along the transverse axis of the cell (Chung et al., 2021) whereas oxidative mouse and human muscles display more grid-like mitochondrial networks (Bleck et al., 2018; Caffrey et al., 2019). However, inconsistencies surrounding the function of mitochondrial subpopulations may be more closely related to experimental procedures. Specifically, the procedures used to isolate mitochondria disrupt critical functional aspects of mitochondrial structure, such as mitochondria-mitochondria contact sites. Furthermore, work from Hoppel and others has revealed key limitations to mitochondrial subpopulation isolation techniques (Kras et al., 2016; Lai et al., 2019) and demonstrated that previous preparations of SSM may have been contaminated with membranous proteins that would have resulted in lower measures of mitochondrial energy conversion. Using a revised protocol, the same group demonstrated that the rates of energy conversion and enzymatic activities are consistent between the IFM and SSM subpopulations, except for SSM having a 10% lower oxygen consumption rate when stimulated with saturating ADP concentrations. This reduction in oxygen consumption capacity in the SSM was also associated with lower cytochrome C content, but it is unclear if the difference in the cytochrome profile between these two mitochondria subpopulations is related to the metabolic or apoptotic functions. Indeed, cytochrome C is associated with mitochondrial apoptotic pathways, and earlier studies have suggested that IFM may be more sensitive to apoptotic stimuli while PM may be more inclined to produce ROS or proton leak (Adhihetty et al., 2005).

Despite these methodological concerns surrounding isolated preparations of mitochondrial subgroups, experiments in isolated mitochondria have produced interesting results when evaluating the apoptotic and ROS-generating capacity of mitochondria. For example, Hood and collaborators demonstrated that IFM isolated from the quadriceps muscles of mice are more sensitive to the apoptotic stimulus hydrogen peroxide compared to SSM (Adhihetty et al., 2005). Although IFM released 10-fold the amount of apoptosis-inducing factor upon exposure to hydrogen peroxide, the same work also demonstrated that SSM produced nearly a threefold greater rate of ROS production compared to the IFM (Adhihetty et al., 2005). Moreover, studies looking at proton leak rate and protein expression have found that IFM may express less uncoupling proteins (UCP3) (Jimenez et al., 2002) and be more efficient (less proton leak) than SSM (Iossa et al., 2001; Mollica et al., 2006). These findings suggest that mitochondrial subpopulations differ remarkably in their capacity to handle oxidative stress and may provide insight regarding the distribution of signaling responsibilities within the mitochondrial reticulum. Specifically, the increased capacity of SSM to generate ROS while also being less prone to ROS-induced apoptosis may indicate that the mitochondria residing within these subgroups are uniquely designed to produce ROS as part of a more integrated cell signaling mechanism rather than isolated local apoptotic pathways. Undoubtedly, mitochondria in PM subgroups are in close proximity to embedded capillaries and peripherally-located myonuclei, and therefore, the reported differences in ROS production and thermodynamic driving forces between mitochondrial subgroups may be associated with proximity to a cellular oxygen supply and/or mitochondria-nuclear signaling (Dominy and Puigserver, 2013; Soledad et al., 2019).

More recent imaging studies have brought some clarity to the diversification of mitochondrial function within living cells by using 3D and 4D imaging strategies to directly evaluate mitochondrial respiratory enzymes, mitochondrial redox, and even the rate of mitochondrial energy conversion within an intact network (Schroeder et al., 2010; Glancy et al., 2015; Willingham et al., 2019). Certainly, spatially resolved measurements within intact cells provide far more specificity regarding the subcellular distribution of mitochondrial heterogeneity compared to studies in isolated mitochondria. For example, imaging studies measuring the spatial distribution of mitochondrial enzymes have found that the membrane potential-generating element (complex IV) and the complex responsible for ATP production (complex V) preferentially localize to mitochondria within the peripheral and intermyofibrillar spaces of the muscle cell, respectively. Specifically, the peripheral localization of Complex IV suggests that PM subgroups may support the IFM by generating proton-motive force and distributing it through the IFM network where it is used to synthesize ATP (Figure 2). Moreover, we recently established a metabolic imaging approach to quantitatively assess mitochondrial function and measure redox kinetics with subcellular resolution (Willingham et al., 2019). The application of this technique in living tissues has provided the first spatially-resolved measure of the rate of *in vivo* mitochondrial energy conversion. Although this work found the energy conversion rate to be consistent across PVM and IFM subpopulations, we found the PVM to be more oxidized than the IFM (Schroeder et al., 2010; Willingham et al., 2019). In keeping with data from isolated mitochondria, these imaging studies demonstrate the functional specialization and differential distribution of thermodynamics across subfractions of the intact mitochondrial reticulum in skeletal muscle cells, but further work is warranted to determine whether subpopulations of muscle mitochondria play distinct roles in other key functions such as Ca^2+^ signaling, apoptosis, fuel importation, and biosynthesis.

## Morphological and Functional Plasticity in Mitochondrial Subpopulations

Subpopulations of mitochondria may also be differentially influenced by exercise training and pathologies. For example, over 40 years ago, a series of imaging studies provided evidence that SSM are more responsive to changes in energetic demand than IFM (Hoppeler et al., 1973a; Muller, 1976) by showing that the relative exercise-induced increases in mitochondrial volume were more significant in the SSM compared to the IFM. In human volunteers, Hoppeler et al. (1973b) found PM to be 3.2-fold higher in exercise-trained muscles compared to untrained while IFM was only increased by 30% with training. These findings have been supported by subsequent experiments in isolated mitochondria that demonstrated SSM experience greater increases metabolic capacity with exercise training compared to IFM. For example, Koves et al. (2005) demonstrated that 10 weeks of exercise training increases fatty acid oxidation ∼100% in SSM but only 50% in IFM, indicating that subpopulations of mitochondria differentially adapt to sustain energy homeostasis in the face of metabolic challenge. Although few studies have compared the function of mitochondrial subgroups in models of skeletal muscle pathology, some experiments in isolated mitochondria have also found that SSM may be more susceptible to dysfunction in the presence of metabolic pathology or inactivation (Kras et al., 2018, 2019). While it is difficult to interpret these findings considering the limitations of isolation techniques, these results are consistent with imaging studies demonstrating that PM subgroups are more affected by pathologies associated with obesity and insulin resistance (Ritov et al., 2005; Nielsen et al., 2010; Chomentowski et al., 2011; Lai et al., 2017). Furthermore, obesity and diabetes are also associated with increased accumulation of lipids within the subsarcolemmal space (Nielsen et al., 2010), but it is unclear how changes in muscle lipid content may influence mitochondria-lipid interactivity, and function, of peripherally-located mitochondria. Changes in muscle metabolic capacity may also be regulated by changes in PM and IFM mitochondrial network structures. While the mechanisms that regulate mitochondrial network configuration in skeletal muscle remain unclear, the overall dynamics of mitochondrial morphology are regulated in part by the balance of fusion/fission events and the cytoskeletal framework (Glancy et al., 2020; Glancy and Balaban, 2021), and pathologies and changes in metabolic demand that influence these key regulators may alter muscle metabolism through changes in mitochondrial network ultrastructure (Shah et al., 2019; Liu et al., 2020). Future studies are needed to establish the link between the subcellular specialization of mitochondria and the functional plasticity of skeletal muscle cells and determine how pathology influences the mitochondria-organelle interactome.

## New Frontiers in Muscle Mitochondrial Biology

Recent advancements in nanoscale imaging technologies have greatly expanded our capacity to investigate subcellular structure, and applications of contemporary techniques have shed new light on mitochondrial biology. Work from Lippincott-Schwartz (Valm et al., 2017), Nunnari (Lewis et al., 2016), and others have used high-resolution live-cell imaging to demonstrate that cellular functions are modulated by a complex network of functional mitochondrial-organelle interactions. In brief, mitochondria form contact sites with the ER, lipid-droplets, vesicles, and the sarcolemma that facilitates a broad range of cellular functions, including metabolism, biosynthesis, apoptosis, and fusion/fission. Although most of the new information regarding functional relationships among subcellular structures has been derived from cell culture models, some studies have found mitochondria-organelle interactions to be associated with functional specialization of mitochondria in the cells of salivary (Porat-Shliom et al., 2019) glands, pancreatic tissues (Johnson et al., 2003), and cardiomyocytes (Lu et al., 2019). While this work suggests that mitochondrial-organelle interactions play key roles in physiological function, further work is needed to characterize the subcellular distribution of mitochondria-organelle interactions in striated muscle cells and determine if differences in organelle interactivity contribute to the specialization of function between the IFM and PM subgroups. Additionally, the *in vivo* functions of many mitochondria-organelle contact sites have not been determined in striated muscle cells, and it is unclear whether or not the molecular architecture of mitochondria-organelle contact sites within skeletal muscle cells is consistent with those observed in cell cultures and other tissues. Future experiments may address these questions by using high-resolution and live-cell imaging strategies to map organelle connectivity across large subcellular volumes and comparing the functional capabilities between subpopulations of mitochondria under different experimental conditions. As discussed, recent advancements in high-resolution 3D imaging and automated image analysis have greatly expanded our capacity to measure the subcellular distribution of mitochondrial morphology and mitochondria-organelle interactivity, but contemporary nanoscale imaging strategies, such as EM, are still limited in their ability to provide molecular and functional information. Therefore, answering the many questions that remain surrounding the subcellular specialization of mitochondrial form and function in skeletal muscle cells will require innovative imaging strategies that evaluate mitochondrial function, composition, and structure at the subcellular level with molecular specificity.

## Author Contributions

All authors listed have made a substantial, direct and intellectual contribution to the work, and approved it for publication.

## Conflict of Interest

The authors declare that the research was conducted in the absence of any commercial or financial relationships that could be construed as a potential conflict of interest.

## Publisher’s Note

All claims expressed in this article are solely those of the authors and do not necessarily represent those of their affiliated organizations, or those of the publisher, the editors and the reviewers. Any product that may be evaluated in this article, or claim that may be made by its manufacturer, is not guaranteed or endorsed by the publisher.
